# The Translational and Rotational Dynamics of a Colloid Moving Along the Air-Liquid Interface of a Thin Film

**DOI:** 10.1038/s41598-018-26121-0

**Published:** 2018-06-11

**Authors:** Subhabrata Das, Joel Koplik, Raymond Farinato, D. R. Nagaraj, Charles Maldarelli, Ponisseril Somasundaran

**Affiliations:** 1 Columbia University, Langmuir Center of Colloids and Interfaces, New York, 10025 USA; 2 City College of The City University of New York, Levich Institute and Department of Physics, New York, 10027 USA; 3 Solvay Technology Solutions, Stamford, USA; 4 City College of The City University of New York, Levich Institute and Department of Chemical Engineering, New York, 10027 USA

## Abstract

This study examines the translation and rotation of a spherical colloid straddling the (upper) air/liquid interface of a thin, planar, liquid film bounded from below by either a solid or a gas/liquid interface. The goal is to obtain numerical solutions for the hydrodynamic flow in order to understand the influence of the film thickness and the lower interface boundary condition. When the colloid translates on a film above a solid, the viscous resistance increases significantly as the film thickness decreases due to the fluid-solid interaction, while on a free lamella, the drag decreases due to the proximity to the free (gas/liquid) surface. When the colloid rotates, the contact line of the interface moves relative to the colloid surface. If no-slip is assumed, the stress becomes infinite and prevents the rotation. Here finite slip is used to resolve the singularity, and for small values of the slip coefficient, the rotational viscous resistance is dominated by the contact line stress and is surprisingly less dependent on the film thickness and the lower interface boundary condition. For a colloid rotating on a semi-infinite liquid layer, the rotational resistance is largest when the colloid just breaches the interface from the liquid side.

## Introduction

**Figure 1:**
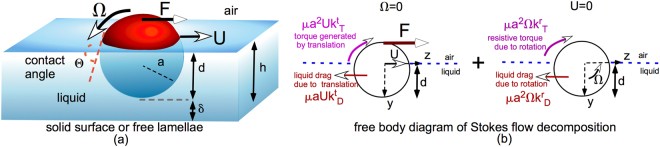
(**a**) Schematic representation of a sphere translating and rotating on the free interface of a thin liquid film on either a solid support or on a free lamellae and (**b**) free body diagram of the decomposition of this motion in Stokes flow.

Colloids which adsorb from a liquid phase onto an air/liquid interface experience a reduction in interfacial energy as the surface area of the colloid, formerly in contact with the liquid, now straddles the surface (see Fig. 1a). This change in interfacial energy is due to the fact that the surface of the colloid is now in partial contact with the gas phase, rather than solely with the liquid, and replaces interfacial water that formerly occupied the attaching position. For a sphere of radius *a*, the change in interfacial free energy (relative to the gas phase) is −π*a*^2^*γ*(1 + cos *θ*)^2^, where *a* is the colloid radius, *γ* is the interfacial tension of the gas/liquid interface, and *θ* is the three phase contact angle measured through the liquid phase. When the colloid surface is only partially wetting (cos *θ* ≠ 1), the energy can be substantially negative and much larger than the thermal energy *k*_*B*_*T* when *a* is order hundreds of nanometers or larger. Hence the colloids become energetically trapped at the interface, while having the freedom to move along the surface in their own version of Abbot’s *Flatland*^1^. Their permanence on the surface armors the interface against coalescence with other surfaces, and generates a resistive surface rheology. Both of these effects play central roles in traditional colloid-related technologies in the chemical, oil and mineral flotation industries as well as in new emerging technologies. For example, nano- and micron sized colloids (10 nm–10 *μ*m) are added to the liquid phases in the preparations of foams and emulsions; the colloids adsorb to the interfaces of the dispersed phase where they stabilize the dispersion from coalescence (i.e “Pickering” emulsions^2^) and thereby increase the lifetime of the dispersion. Water droplets naturally occur in crude oil recovered from reservoirs, and the droplets are stabilized from coalescence by the adsorption onto the droplet interfaces of clay colloids (100 nm–10 *μ*m) found in the crude. A fundamental challenge in hydrocarbon processing is to remove these stabilized droplets which typically do not coalesce to larger droplets and sediment out. In the process of flotation, minerals (order 100 *μ*m) suspended in a liquid phase are removed by adsorption to rising gas bubbles which are collected in a surface foam^3^.

In most of the above applications, the fluid interface on which the colloids are located separate a droplet phase from a continuous phase, and the thickness of both adjoining phases are large relative to the size of the colloid. The goal of this study is to examine the case in which the thickness of the fluid phases which bound the interface can become of the order of the colloid size. We particularly focus on colloids straddling the air/liquid interface of a planar thin liquid film and examine the cases in which the opposing film interface, opposite to the interface that attaches the colloid, is either a solid substrate or a free liquid film (see Fig. 1a). Colloids attached to the interfaces of thin lamellae occur in many applications: In Pickering foams and emulsions, colloids attached to thin liquid lamellae arise when the continuous phase volume fraction becomes small (e.g.“dry” foams). Flotation processes also involve conditions in which the liquid fraction is small. New, emerging technologies aim to assemble colloids straddling the surface of a liquid film on a solid support into well-packed or even crystalline domains, or into programmed assemblies to be used as coatings (superhydrophobic or antireflection), templates for the materials fabrication of micro and nano-structured materials, photonic crystals and for optoelectronic devices^4,5^. In these technologies, thin liquid films containing suspensions of colloids are spread onto a solid substrate; as the colloids adsorb onto the surface, the subsequent evaporation of the film draws the colloids together by capillary forces to create assembled structures. Magnetic and electrostatic forces applied to magnetized or charged colloids, respectively, are also used to tune the assembly which can further be tuned by using active colloids which propel themselves^6^.

When colloids are attached to the interface of a thin liquid film (either on solid supports or with a free boundary), their hydrodynamics become complicated as they translate and rotate along the surface because the colloid hydrodynamically interacts with the opposite surface (Fig. 1a). The presence of these bounding surfaces changes the resistive shear stresses exerted on the colloid by the fluid, which in turn affects the colloid’s translational and rotational velocities. Several studies, using a continuum framework, have examined the translation of a spherical colloid along a fluid interface separating a gas phase from a semi-infinite, Newtonian liquid phase in a flow regime where inertial effects are negligible, i.e. the Reynolds number, ${Re}=\rho Ua/\mu \ll 1$ where *a* is the colloid radius, *U* the velocity, *μ* and *ρ* the liquid viscosity and density respectively. The interface on which the colloids are confined is assumed to be flat (with the contact line perfectly circular, Fig. 1a). In fact, a colloid can locally deform the interface if it is acted on by a force normal to the interface, e.g. gravity which can create a depression (or elevation) for a colloid with density *ρ*_*s*_ larger (smaller) than the liquid. The fluid interface can also deform due to the viscous fluid stresses which develop in the underlying liquid as the colloid moves along the surface^7^. In either case, the interfacial deformation is resisted by the restoring force of the surface tension, *γ*. If surface tension forces are large enough relative to the normal force (Bond number, $Bo=|{\rho }_{s}-\rho |g{a}^{2}/\gamma \ll 1$) or viscous forces (capillary number, $Ca=\mu U/\gamma \ll 1$) deforming the interface, the surface remains approximately flat up to the three phase contact line. In this case, the immersion depth is then only given by the contact angle (Fig. 1a, *d*/*a* = 1 + cos(*θ*)). Under the above two assumptions, the equations for the colloid motion are linear (Stokes flow) and the flow can be divided into two realizations (Fig. 1b) - one in which the the colloid is moving under an applied lateral force but not rotating, and the second in which the colloid is rotating but not translating.

While the restriction that the capillary number is small does not involve the diameter, the requirements that the Bond and Reynolds numbers be small restricts the colloid size. For typical values for the translational velocity *U* of order of 10^−3^ ms^−1^ (values measured for capillary attraction^8–12^), colloid densities of order 10^3^–10^4^ kg m^−3^, liquid density and viscosity of order 10^3^ kg m^−3^ and 10^−3^ kg m^−1^ s^−1^, respectively, and surface tension of the order of 10 mNm^−1^, colloid diameters should be less than approximately 10 *μ*m for *Bo* < 10^−2^ and *Re* < 10^−2^ which infers that the colloid size range which satisfies the restrictions of our study approach the colloid domain. For nanoparticles, the diffusion and forced motion of nanoparticles on fluid interfaces have been studied theoretically using molecular dynamics simulations^13,14^ without gravity and low to order one Reynolds number, and noticeable meniscus curvature at the contact line was not observed, either as the colloids diffused along the surface or were forced to move by an external force.

Past studies using either boundary integral^15,16^, finite element^17,18^ or integral transform methods^12,19^ have focused on the translation, and have assumed that the colloid does not rotate due to the presence of surface roughness or chemical heterogeneities that pin the three-phase contact line (contact angle hysteresis). However recently, evaluating the drag exerted on non-smooth colloids^14^ with heterogeneous surfaces as they diffuse along an interface while specifically examining the role of contact line pinning and de-pinning have been important given the extensive interest in particle-laden interfaces. For the translational motion with velocity *U*, the fluid exerts a drag in the opposite direction to the motion calculated in terms of a drag coefficient ${k}_{D}^{t}$ (i.e. the drag force is $\mu a{k}_{D}^{t}U$) as a function of the immersion depth of the colloid into the liquid (*d*). The drag coefficient increases as the colloid becomes more immersed in the liquid phase, but the colloid is required to be of order ten diameters away from the interface before the drag coefficient of a colloid in an infinite medium is obtained. The translational motion of the colloid along the interface in the positive *z* direction also generates a torque along an axis parallel to the interface and perpendicular to the flow (Fig. 1b) which acts to rotate the colloid counterclockwise. This torque arises because the shear stress exerted by the liquid on the underside of the colloid as it moves is much larger than the torque exerted by these stresses on its topside due to the fact that part of the top is exposed to the vapor. The coefficient for the generated torque ${k}_{T}^{t}$ (the torque is $\mu {a}^{2}{k}_{T}^{t}U$) has also been calculated, which at first increases as the colloid breaches the interface from the vapor side, achieves a maximum when the immersion depth is one colloid radius (*d*/*a* = 1) and the stress asymmetry is the largest and then decreases to zero as the colloid becomes further immersed into the liquid phase. The drag coefficient due to translation has been used to model the pairwise interaction of two colloids atop an air-semi-infinite liquid phase at a particular immersion depth, and subject to capillary attraction forces due to the overlap of the interfacial depressions of the interface, and the theoretical predictions are in agreement with the measurements^8–12^. These results have been extended for the case of flow over a monolayer of interfacial colloids at the liquid-gas interface^20^.

If the force acting on the colloid is large enough, the torque generated by the motion can overcome the pinning forces and the colloid is able to rotate. This rotation has only been studied for the case in which half of the colloid is immersed in the liquid phase (*d*/*a* = 1 or *θ* = *π*/2) by Brenner *et al*.^21,22^ who develop an eigenfunction solution for this immersion depth. Brenner *et al*. pointed out that the rotation requires the meniscus at the three phase contact line to advance (along one face) and recede (along the opposite face) along the surface of the colloid. As the liquid advances or recedes, a non-integrable liquid shear stress develops on the colloid surface due to the imposition of the no-slip condition on the solid surface because the surface velocity at the contact line is discontinuous as it is approached from the solid/liquid and gas/liquid sides. This results in the non-physical conclusion that the colloid cannot rotate due to this non-integrable stress. This contact line singularity due to no-slip can be removed by the inclusion of several physicochemical hydrodynamic effects^23^: (i) Inclusion of slip at the contact line by a Navier slip condition, (ii) the introduction of intermolecular (disjoining pressure) forces in the fluid in the vicinity of the contact line which can lead to precursor films ahead of the macroscopic contact line, (iii) modelling the contact line motion as a thermally activated process using molecular kinetic theory in which progression of the contact angle is in steps that overcome energetic barriers and (iv) the inclusion of evaporation and condensation of liquid at the contact line, allowing the liquid to move over an existing thin layer. Here, the Navier slip model is used as experiments have revealed the presence of small slip coefficients (order 1–10 nm) in the motion of liquids over solid surfaces which are smooth on the scale of order tens of nanometers or smaller^24–27^. Brenner *et al*. use the Navier slip model to account for slip at the contact line. For a colloid rotating about the x-axis with angular speed Ω (Fig. 1b), if the dimensional velocity of the fluid at the colloid surface is denoted by ${{\rm{v}}^{\prime} }_{\varphi ({\rm{s}})}$, where *ϕ* is the azimuthal angle about the x axis, and the dimensional viscous fluid stress on the colloid in the *ϕ* direction by ${\tau ^{\prime} }_{{\rm{r}}\varphi ({\rm{s}})}$ (where *r* is the radial spherical coordinate from the sphere center), the Navier slip condition is of the form $({{\rm{v}}^{\prime} }_{\varphi ({\rm{s}})}-{\rm{\Omega }}a)=\frac{\lambda }{\mu }{\tau ^{\prime} }_{{\rm{r}}\varphi ({\rm{s}})}$ where *λ* is the slip coefficient. As *λ* → 0, the no-slip condition is recovered. Closed form solutions for the translational coefficients were obtained, ${k}_{D}^{t}=3\pi \frac{1+2\lambda \,/\,a}{1+3\lambda \,/\,a}$ and ${k}_{T}^{t}=\frac{3\pi }{2}\frac{1}{1+3\lambda \,/a}$. For the rotational problem, a counterclockwise rotation of the colloid generates a shear stress on the colloid which acts to drag the fluid in the −*z* direction, as well as create a resistive torque (directed clockwise), cf. (Fig. 1b). The drag force is given by $\mu {a}^{2}{k}_{D}^{r}{\rm{\Omega }}$ where ${k}_{D}^{r}$ is the drag coefficient due to rotation, and Brenner *et al*. showed that this coefficient is equal to ${k}_{T}^{t}$ as required by the reciprocity theorem. Note all these coefficients are bounded as *λ* → 0, when the no-slip condition prevails, and hence the rotation of the colloid does not create a singularity in the drag. The resistive torque due to the rotation, ${k}_{T}^{r}$ (the torque is $\mu {a}^{2}{k}_{T}^{r}a{\rm{\Omega }}$), was computed by series summation as a function of *λ* diverges as the slip length decreases to zero owing to the contact line singularity with a correlated logarithmic divergence (${k}_{T}^{r}={K}_{1}\ell n(a/\lambda )+{K}_{2}$ as *λ* → 0 with *K*_1_ ≈ 4.5).

Numerical solutions are developed for the translation and rotation of a colloid moving on the surface of a planar thin film which is bounded from below by a solid wall or a free surface, accounting for both the interfacial motion (as in the semi-infinite studies) and the colloid-lower surface hydrodynamic interaction (as in the studies in which the colloid is fully immersed and in proximity to an adjacent no-slip or zero-stress interface). As in the earlier studies, inertialess flow and a flat interface with a circular contact line will be assumed. Both the translational and rotational parts of the problem are being addressed. To alleviate the contact line singularity and allow the colloid to rotate, the Navier slip condition is used on the colloid surface with a small slip coefficient *λ*/*a* that we fix at 0.01 which would be characteristic for a 1 *μ*m colloid and a slip length of 10 nm. Numerical solutions for the drag and torque coefficients for translation and rotation are obtained using a finite element solver (COMSOL Multiphysics) (see Methods and Supplementary Information). Similar finite element models (FEM) using COMSOL Multiphysics has been implemented to solve Stokes equations for a sphere in bulk of a rectangular channel under Poiseuille flow conditions^28^. The cases of a film on a solid support, and on a free-interface are being separately studied, and in both the effect of the film thickness *h*/*a* is considered, with the contact angle being that of a neutral wetting colloid (cos(*θ*) = 0, d/a = 1). Finally, a more detailed study is given on the rotation of a colloid on the surface of a semi-infinite liquid, and the rotational coefficients are computed as a function of the immersion depth. The discussion uses the drag and torque coefficients to detail sliding and rotating regimes for a colloid moving under a constant force applied along the interface of a free film, and a film on a solid support.

## Results

### Hydrodynamics of a Sphere on the Interface of a Thin Film Resting On a Solid Surface

**Figure 2:**
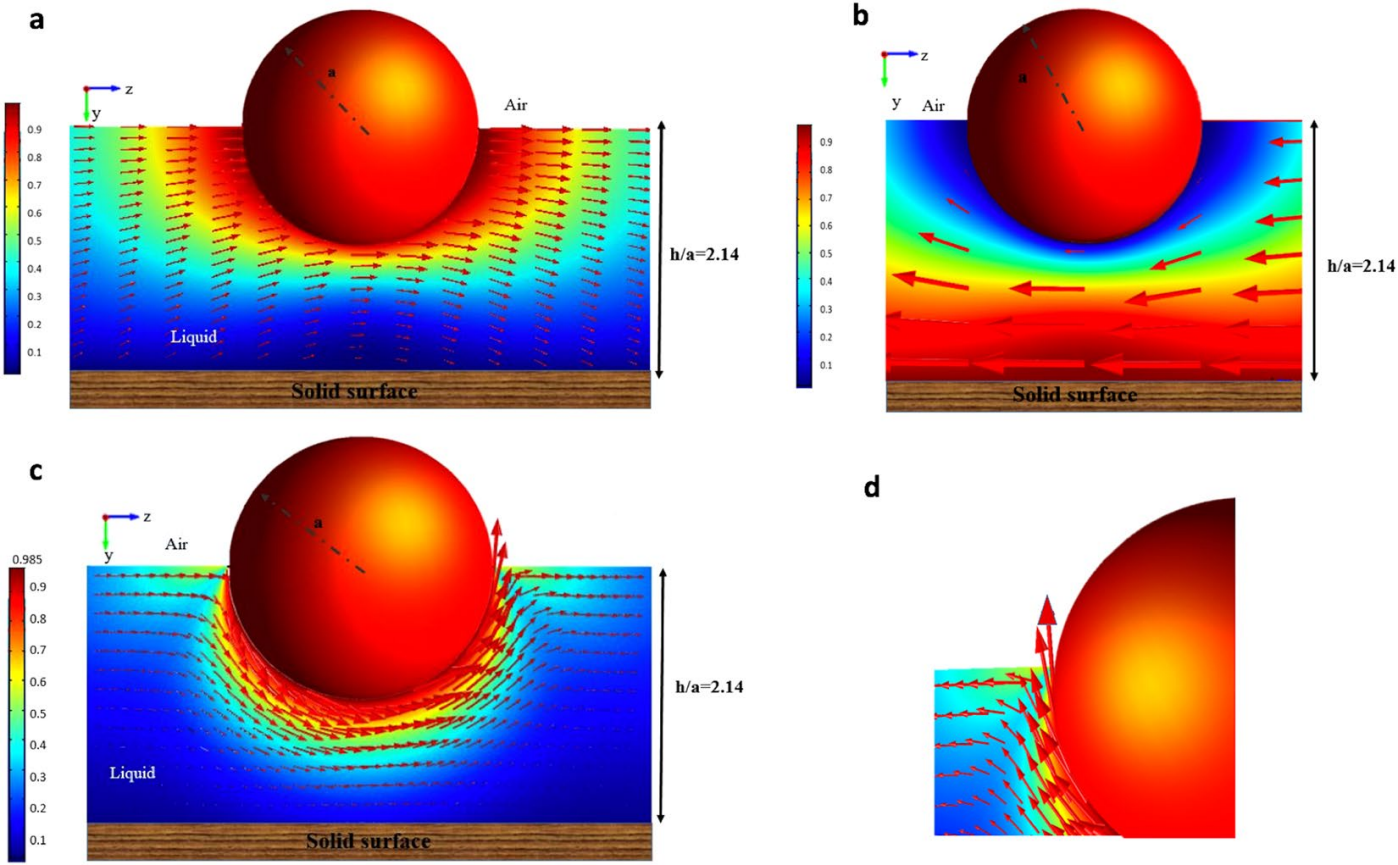
Streamlines and map of the fluid velocity in the *x* = 0 symmetry plane of the flow in the thin liquid layer underneath a colloid translating in the positive *z* direction with velocity *U* along the film surface in (**a**) the lab frame in which the colloid is translating, and (**b**) in the colloid frame in which the liquid is approaching the stationary sphere in the −*z* direction. (**c**) Streamlines and map of the fluid velocity in the *x* = 0 plane of the flow in the liquid layer underneath a sphere rotating counterclockwise around the *x* axis with velocity Ω*a* at the interface of the film for (a) the entire film and (**d**) in the immediate vicinity of the contact line. The thickness of the layer is *h*/*a* = 2.14, the contact angle is *π*/2 and the slip length *λ*/*a* is 0.01.

In this section, the hydrodynamic drag and torque coefficients exerted on a translating and rotating colloid straddling an air/liquid interface above a thin liquid film on a solid substrate are being calculated. Starting with the kinematics, Fig. 2a gives an illustration of the flow field around a colloid translating along the interface with velocity *U* in the *z* direction in the reference frame in which the substrate is stationary. The thickness of the film relative to the colloid radius is *h*/*a* = 2.14 (so that film thickness is slightly large than the colloid diameter 2*a*) and contact angle *θ* = *π*/2. The slip length at the colloid surface *λ* divided by the colloid radius *a* is 0.01 $(\frac{\lambda }{a}=0.01)$. The figure shows the fluid velocity vectors in the stationary frame of the substrate, in a plane (the *y*–*z* plane) which is perpendicular to the substrate and cuts the sphere in half. Hence in this plane the flow field is symmetric with respect to *x*, and the velocity in the *x* direction is equal to zero. The displayed magnitude of the vectors are proportional to the magnitude of the velocity. The color scheme in the figure provides the magnitude of the velocity in the *y*–*z* plane scaled by the colloid velocity U, i.e. ${[{{\rm{v}}}_{z}^{2}+{{\rm{v}}}_{y}^{2}]}^{1/2}/U$. The flow shows fore-aft symmetry about *z* = 0 because the flow Reynolds number is very small (creeping flow). The surface flow at the gas/liquid interface is evident. The upward and downward shapes of the flow streamlines aft and forward of the moving sphere is due to the forward motion of the sphere, and the fact that the free interface is required to remain flat. So, fluid is drawn up aft of the sphere, and is pushed down in the forward direction. The flow field in the reference frame of the moving colloid is shown in Fig. 2b. In this case, as expected, liquid flows around the stationary colloid, moving downward on the upstream side of the colloid, and moving upward on the downstream side. Adding to the velocity field in this reference frame a uniform motion in the *z* direction yields the flow field depicted in Fig. 2a.

The flow streamlines for the case in which the colloid is rotating (but not translating) counterclockwise about the *x* axis with angular velocity Ω in the symmetry plane *x* = 0 is given in Fig. 2c,d in the lab fixed frame, for the same values used for translation, i.e. *θ* = *π*/2, *h*/*a* = 2.14 and *λ*/*a* = 0.01. (As in Fig. 2a, displayed velocity vectors and the magnitude of the velocity are all scaled with Ω*a*). The figure makes clear that the disturbance flow caused by the rotation is more localized around the sphere than in the case of the translating colloid (compare Fig. 2a,c). This can be understood from the difference between the decay of the fluid velocity for rotating and translating spheres in an infinite medium, where the velocity decays as the inverse of the distance from the sphere center (i.e. 1/*r*) for the translating case, and 1/*r*^2^ for the rotating case. Of more interest is the flow detail in the immediate vicinity of the contact line (Fig. 2d) where the velocity (scaled by the non-dimensional velocity of the rotating sphere, Ω*a*) has to sharply change direction from normal to tangential to the surface. This change gives rise to large velocity gradients in the immediate vicinity of the contact line. Note that the magnitude of the fluid velocity at the sphere surface in the contact line vicinity is much smaller than the velocity of the rotating sphere itself. However, further away from the contact line (i.e. towards the bottom of the colloid), the velocity of the fluid in the immediate vicinity of the rotating sphere is much larger and equals the rotating velocity of the sphere. The reason is due to the large shear stresses on the rotating colloid at the contact line. These large stresses give rise at the corner to a difference between the fluid velocity adjacent to the rotating colloid and the velocity of the colloid surface itself (slip) in accordance with the Navier slip condition $({{\rm{v}}^{\prime} }_{\varphi ({\rm{s}})}-{\rm{\Omega }}a)=\frac{\lambda }{\mu }{\tau ^{\prime} }_{{\rm{r}}\varphi ({\rm{s}})}$ where ${{\rm{v}}^{\prime} }_{\varphi ({\rm{s}})}$ is the (dimensional) rotational fluid velocity (about the *x* axis) at the surface and ${\tau ^{\prime} }_{r\varphi (s)}$ is the (dimensional) shear stress exerted by the liquid on the sphere in the rotating (*ϕ*) direction. The velocity difference is proportional to the slip coefficient multiplied by the stress, and even though the slip coefficient in the simulations is small (*λ*/*a* = 0.01) the large stresses account for the observable slip. Away from the contact line, but still in the vicinity of the surface, the shear stresses are smaller and the fluid hardly slips. This accounts for the higher velocities observed for the fluid in the immediate vicinity of the rotating colloid and away from the contact line.

**Figure 3:**
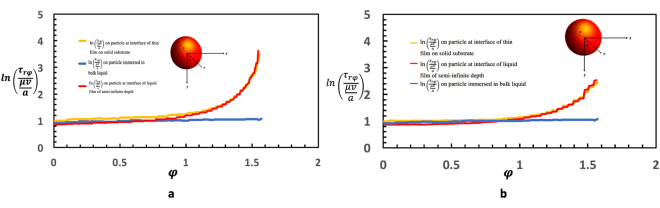
Shear stress distribution (${\tau ^{\prime} }_{r\varphi }$) along the sphere surface in the plane *x* = 0 as a function of *ϕ* measured from the bottom pole of the colloid for a colloid with *d*/*a* = 1 on the surface of a thin film above a solid (*h*/*a* = 30), above a semi-infinite liquid (*h*/*a* = 2.14) and when completely immersed in an infinite medium. (**a**) *λ*/*a* = 0.01 (**b**) *λ*/*a* = 0.05.

The shear stress (${\tau ^{\prime} }_{r\varphi (s)}$) in the plane *x* = 0 exerted on the rotating colloid (scaled by *μ*Ω) is plotted in Fig. 3a as a function of the angular position *ϕ* along the surface measured from the bottom pole of the colloid. Because of the symmetry about the *x* = 0 plane, this is the only component of the shear in this plane and it is clear that as the three phase contact line is approached (*ϕ* = *π*/2) the stress increases by two orders of magnitude relative to the value at the bottom of the sphere (i.e. nearest to the wall (*ϕ* = 0)). This increase reflects the large velocity gradients at the contact line region that creates a large viscous dissipation. In contrast, also plotted in the figure is the shear stress for the case in which the sphere is totally immersed in the liquid phase and ${\tau ^{\prime} }_{r\varphi (s)}$ is nearly uniform, as required by symmetry, and the average value (2.75 *μ*Ω) compares to the theoretical value $3\mu {\rm{\Omega }}\frac{1}{1+3\lambda /a}=2.97\,\mu {\rm{\Omega }}$ for *λ*/*a* = 0.01. (The values in the figure for this completely submerged case correspond to an immersion depth of *d*/*a* = 15, and a film thickness *h*/*a* = 30; at these values, the effect of the free interface and the solid wall on the rotation are negligible). Also plotted in Fig. 3a is the shear stress profile (${\tau ^{\prime} }_{r\varphi (s)}(\varphi )$) for the case in which the solid substrate is infinitely far away. (Numerically, this is achieved for *h*/*a* = 15.0; for larger values the stress profile changes by less than one percent). From the figure, it is clear that the stress distribution is slightly less relative to the case of a finitely thick film (*h*/*a* = 2.14) in the vicinity of the wall, but is approximately equal as the contact line is approached. The larger value near the wall reflects the small (1/*r*^2^) interaction of the rotating surface with the wall. However, as the contact line is approached, and the stress becomes elevated due to the large velocity gradients, the effect of the wall becomes negligible and the stress is determined by these large velocity gradients. When the slip coefficient is increased, the shear stress at the contact line is reduced due to the increased slip at the surface of the rotating colloid. In Fig. 3b, ${\tau ^{\prime} }_{r\varphi }$ is plotted as a function of *ϕ* for *λ/a* = 0.05, and note that the increase in slip by a factor of five from *λ*/*a* = 0.01 (Fig. 3a) results in an decrease in the maximal stress at the contact line by the same factor. However, note that the increase in slip from *λ*/*a* = 0.01 to 0.05 results in a decrease in the shear stress only in the vicinity of the contact line; away from the contact line (0 < *ϕ* < 1.4) the shear stresses are identical.

**Figure 4:**
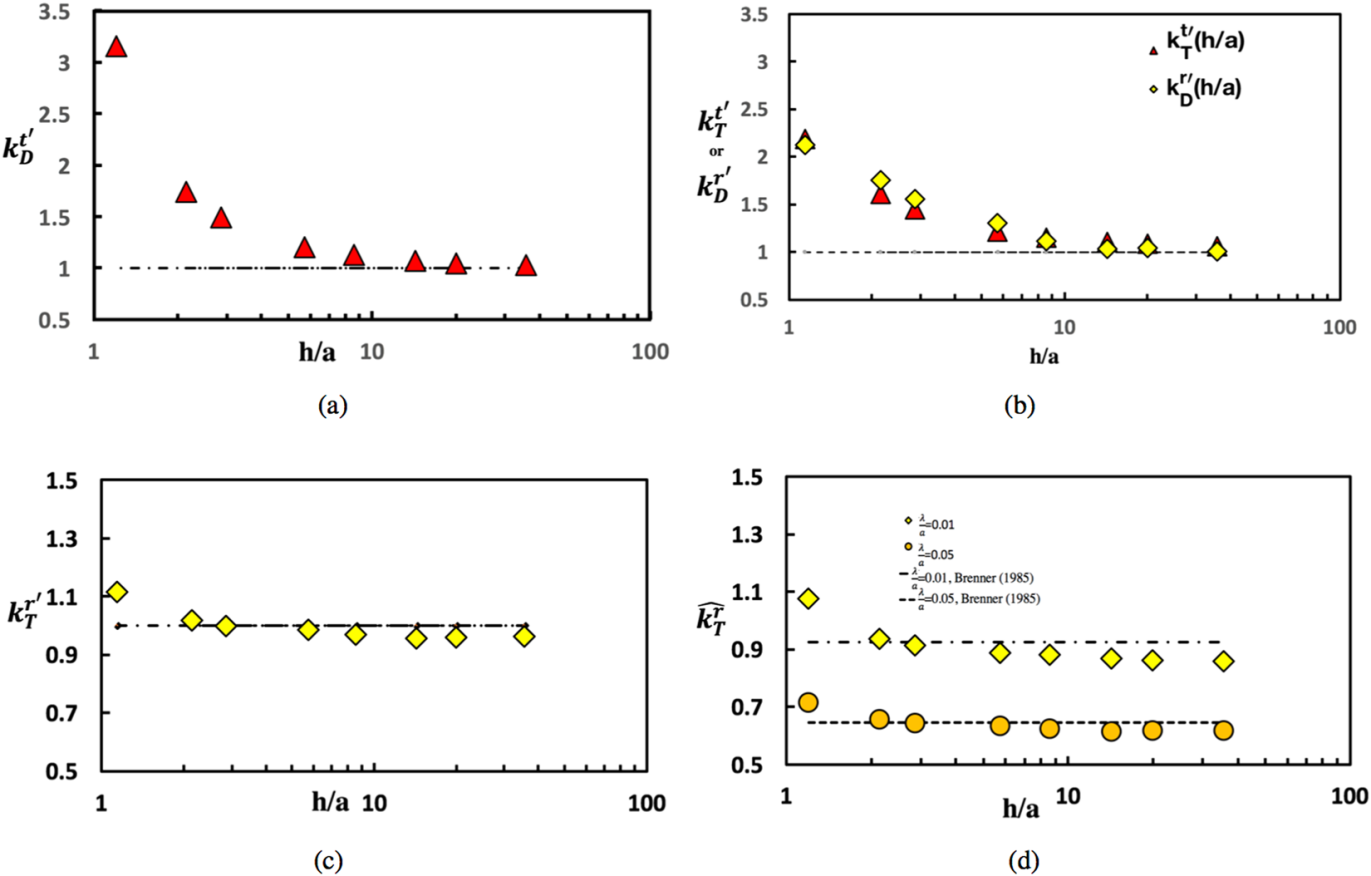
Normalized drag and torque coefficients for colloid motion on the surface of a thin film atop a solid substrate: (**a**) drag coefficient (${k}_{D}^{t\text{'}}$) for a translating colloid, (**b**) coefficient for the torque generated by a translating colloid (${k}_{T}^{t\text{'}}$) or drag coefficient generated for a rotating colloid (${k}_{D}^{r\text{'}}$), (**c**) coefficient of resistive torque (${k}_{T}^{r\text{'}}$) for a rotating colloid all as a function of film thickness and slip length equal to 0.01 and normalized by value atop a semi-infinte medium, and (**d**) Normalized coefficients of resistive torque (${\hat{k}}_{T}^{r}$, scaled by value completely immersed in an infinite medium) as function of film thickness for a rotating colloid for two different slip lengths *λ*/*a* = 0.01 or 0.05 as a function of thickness. The contact angle is *π*/2.

From the solutions for the velocity field for pure translation (along the surface) or pure rotation (around the *x* axis parallel to the surface), the hydrodynamic drag and torque exerted by the viscous liquid film on the colloid due to the liquid stresses generated by the motion are being computed. In the case of translation in the *z* direction (Fig. 1b), the resistive drag force in that direction ($\mu aU{k}_{D}^{t}$) is being normalized by the value of the drag when the underlying phase is semi-infinite. Due to the symmetry of the flow imposed by the zero stress condition at the free surface (*τ*_*yx*_ = *τ*_*yz*_ = 0) the drag on a colloid with *d*/*a* = 1 (*θ* = *π*/2) is exactly half that of the translational drag in an infinite medium, or $3\pi \mu aU\frac{(1+2\lambda /a)}{(1+3\lambda /a)}$ (correcting for the finite slip). This normalized drag coefficient ${k}_{D}^{t^{\prime} }$ as a function of the liquid film thickness *h*/*a* is being plotted in Fig. 4a. As expected, as the film thickness *h*/*a* decreases, the drag increases due to the stronger hydrodynamic interaction with the solid substrate as the dominant resistance is the shear stress exerted on the colloid by the liquid. As the gap between the colloid and the substrate becomes very small, the drag increases dramatically due to the strong viscous lubrication forces in the intervening layer^29^. For Fig. 2a, for which case *d*/*a* = 1, this dramatic increase begins at *h*/*a* ≈ 3 which represents a gap of one colloid diameter. For larger values of the thickness, the interaction with the wall disappears, and the normalized drag approaches the value for the drag for a semi-infinite medium, i.e. one due to the normalization.

As noted in the Introduction, the colloid translates along the surface, the shear stress exerted on the colloid by the shear flow in the underlying liquid (Fig. 1b) exerts a torque on the colloid which acts to rotate the colloid in the clockwise direction around the *x* axis if the translation is in the positive *z* direction. In an infinite medium translation does not give rise to a net torque because of the symmetry of the shear stress around the colloid. But in the case of the colloid straddling an air/liquid interface, the viscous stress in contact with the liquid is not compensated by any shear in the air phase and consequently a net torque is exerted on the floating colloid. The normalized torque coefficient ${k}_{T}^{t^{\prime} }$ is being defined as the torque exerted on the floating colloid divided by the torque on the colloid in the case in which the film is infinite in extent (*h*/*a* → ∞), i.e. the colloid translates over an infinitely thick film. The latter torque was computed by Brenner *et al*.^21^, who calculated it analytically using series solutions of the Stokes equations in spherical coordinates (only appropriate for *d*/*a* = 1) as a function of the slip length *λ*. Figure 4b plots the normalized coefficient as a function of *h*/*a* (normalized by the Brenner *et al*. value), and see that the torque due to the translation increases as the film thickness decreases due to the increase in the shear stress as the translating colloid becomes closer to the wall due to the larger velocity gradients which develop as the translating colloid comes in proximity to the wall.

In the case of pure rotation of the colloid at the surface (about the *x* axis parallel to the interface, see Fig. 1b) the resistive torque exerted by the shear stress in the liquid due to the colloid rotation is being computed, and normalized with the torque exerted in the case in which the liquid layer is semi-infinite and the immersion depth *d*/*a* = 1 (neutral wetting). The latter value was computed by Brenner *et al*.^21^ for *d*/*a* = 1 and arbitrary *λ*/*a*, and is used for the normalization. Plotted in Fig. 4c is the normalized coefficient ${k}_{T}^{r^{\prime} }$ as a function of *h*/*a* and note that although an increase in the normalized coefficient is observed, the increase is much smaller than the increase in the normalized drag coefficient ${k}_{D}^{t\text{'}}$. The reason, as shown in Fig. 3a for *h*/*a* = 2.14, is that most of the resistance to the rotation arises from the corner of the three phase contact line where the shear stress is the largest due to the large velocity gradients. Note that in the vicinity of the contact line this large shear stress is essentially the same for both the case of a finite liquid layer (*h*/*a* = 2.14) and a semi-infinite layer. Away from the contact line and in the vicinity of the wall, the surface shear stress is slightly larger than the value for the semi-infinite case due to the hydrodynamic interaction with the wall. This small increase accounts for the increase observed in the torque coefficient. When the slip on the surface is increased from *λ*/*a* = 0.01 to *λ*/*a* = 0.05 and the stress at the surface of the rotating sphere is consequently reduced in the vicinity of the contact line (compare Fig. 3a,b), the resistive torque due to the rotation decreases. The torques due to rotation for *λ*/*a* = 0.05 and 0.01 in Fig. 4d are being plotted where each is normalized by the torque due to rotation in an infinite medium (i.e. 8*πμa*^3^Ω). Although the shear stress has decreased in proportion to the increase in the slip, the torque only decreases logarithmically as the increase in the shear is restricted to the vicinity of the contact line. This logarithmic dependence on the slip coefficient was also recognized by Brenner *et al*.^21^ for the case of a colloid rotating at an interface for *d*/*a* = 1 where they show that ${k}_{T}^{r}\simeq {K}_{1}\,{\rm{l}}{\rm{n}}\,(a/\lambda )+{K}_{2}$ where ${K}_{1}\simeq 4.5$ and ${K}_{2}\simeq 2.5$.

The resistive stress exerted on the surface of the floating colloid as it rotates counterclockwise around the *x* axis generates a torque which not only resists the rotation, but also causes the colloid to translate along the interface in the negative *z* direction (Fig. 1b). This viscous force for a colloid rotating on the surface of a film of a given thickness *h*/*a* with an immersion depth *d*/*a* = 1 is being calculated, and normalized with the corresponding value when the underlying liquid is semi-infinite (again as determined by Brenner *et al*.^21^). This normalized coefficient, ${k}_{D}^{r\text{'}}$, is plotted as a function of *h*/*a* in Fig. 4b. The coefficient increases rapidly with a decrease in the film thickness when the gap thickness becomes of the order of the colloid diameter (*h*/*a* ≈ 3) due to the large velocity gradients which develop in the gap between the rotating sphere and the wall. More importantly, Fig. 4b shows that for the same value of the film thickness, the cross coefficients of the torque due to translation (${k}_{T}^{r\text{'}}$), and the drag due to rotation (${k}_{D}^{r\text{'}}$) are equal as required by the reciprocity relations.

### Hydrodynamics of a Sphere On the Interface of a Thin Lamella

Similar to the above, the hydrodynamic drag and torque exerted on a translating and rotating spherical colloid straddling an air/liquid interface on a thin liquid film bounded by a free interface on the other side e.g. a foam lamella has been calculated. Because the opposite interface is stress free, the hydrodynamic interaction of the colloid with the opposite film face is weaker relative to the case of a film on a solid substrate. As will become apparent, the presence of a bounding stress-free interface allows the colloid to translate and rotate with a hydrodynamic resistance which is reduced even with respect to the translation or rotation of a colloid on an interface above a semi-infinite liquid, and this resistance decreases considerably as the film becomes thinner.

**Figure 5:**
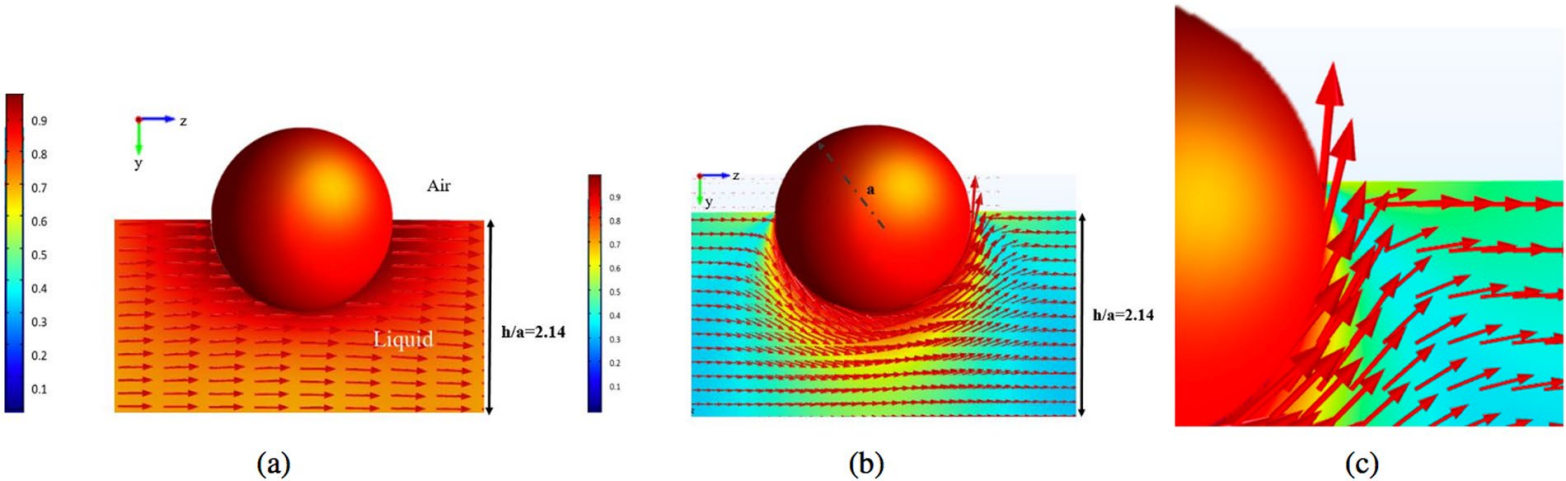
(**a**) Sketch of streamlines and map of the fluid velocity in the *x* = 0 symmetry plane of the flow for a sphere translating in the positive *z* direction with velocity *U* along the interface of a thin film with free interfaces on both sides, (**b**) streamlines and map of the fluid velocity in the *x* = 0 symmetry plane of the flow around a sphere rotating counterclockwise (around the *x* axis with velocity Ω*a*) at the interface of a thin film bound by free interface and (**c**) enlargement of the flow streamlines in the immediate vicinity of the contact line. The thickness of the layer is *h*/*a* = 2.14, the contact angle is *π*/2 and the slip length *λ*/*a* is 0.01. Displayed velocity vectors and the magnitude of the velocity are all scaled with Ω*a*.

Figure 5a shows the flow field in the symmetry plane around a colloid translating with velocity *U* in the *z* direction in the reference frame in which the liquid is stationary, for the same values of the film thickness (*h*/*a* = 2.14), contact angle (*θ* = *π*/2) and slip coefficient (*λ*/*a* = 0.01) as in the case of the solid support. Compared to the flow over a solid substrate (Fig. 2a), no sharp velocity gradients are observed on the underside of the colloid, as the bottom air/liquid interface now moves with a velocity approximately 3/4 of the colloid velocity. Hence the lower boundary does not contribute a strong frictional resistance. In addition, because of the greater mobility of the lower interface, the upward and downward shapes of the flow streamlines fore and aft of the colloid are almost negligible.

The flow streamlines for the case in which the colloid is rotating (but not translating) about the *x* axis with angular velocity Ω in the symmetry plane *x* = 0 is given in Fig. 5b in the lab fixed frame, for the same values used for translation, i.e. *θ* = *π*/2, *h*/*a* = 2.14 and *λ*/*a* = 0.01. As with case of translation of the colloid along the surface of the lamella, no large velocity gradients develop as the bottom free interface moves with a velocity approximately one-half of the velocity of the rotating sphere. The detail of the flow at the three phase contact line (Fig. 5c) is similar to that for a sphere rotating at the interface of a film on a solid support, confirming that the details of the flow at the contact line are not significantly affected by the mobility of the lower interface of the film. However, the velocity gradients at the contact line are slightly smaller relative to the case in which the sphere rotates on a film above a solid support (compare Figs 2d and 5c).

**Figure 6:**
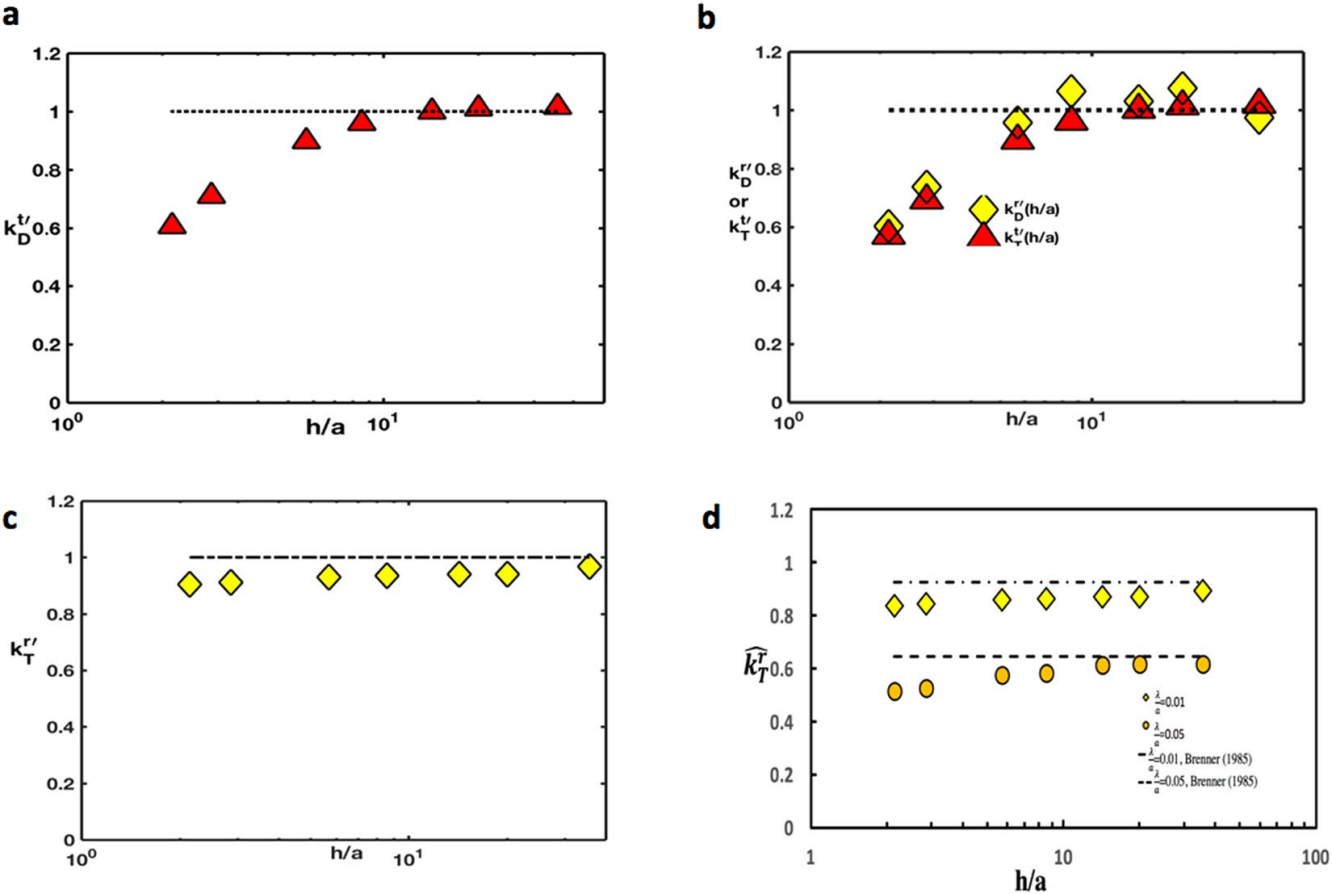
(**a**) Normalized drag coefficient (${k}_{D}^{t\text{'}}$) as function of film thickness for a translating sphere along the interface of a free lamellae, (**b**) normalized cross coefficients as function of film thickness for either a translating or rotating sphere above a free lamellae and (**c**) normalized resistive torque coefficient (${k}_{T}^{r\text{'}}$) as function of film thickness for a rotating sphere on a free liquid lamellae. The contact angle is *π*/2 and the slip length *λ*/*a* is 0.01. (**d**) Normalized coefficients of resistive torque as function of film thickness for a rotating sphere atop a free lamellae for two different slip lengths *λ*/*a* = .01 and 0.05. The contact angle is *π*/2.

The normalized drag coefficient for translation (${k}_{D}^{t}$), and the normalized resistive torque due to rotation (${k}_{T}^{r}$), both normalized by the values for motion and rotation above a semi-infinite liquid, are reported in Fig. 6a,c as a function of *h*/*a* for *λ*/*a* = 0.01 and *θ* = *π*/2. Both coefficients show a *reduction* in hydrodynamic resistance relative to the semi-infinite case because of the presence of the stress free interface and absence of any strong viscous lubrication forces in the gap between the colloid and the bottom interface. In the case of the drag coefficient the reduction is significant and becomes more pronounced as the film thins. The percent decrease is approximately the same as the percent increase in drag for the translation on a film over a solid substrate. However, in the case of the resistive torque, the decrease in the hydrodynamic friction (relative to the semi-infinite value) with a decrease in *h*/*a* is small as this resistance is dominated by the stresses at the contact line. This is similar to the case for the rotation of a colloid on the surface of a thin film above a solid substrate, where the increase in the resistive torque with *h*/*a* is again marginal because of the dominance of the contact line stresses. Figure 6d gives the resistive torque (*θ* = *π*/2) as a function of *h*/*a* for two values of *λ*/*a* (0.01 and 0.05), normalized by the bulk value (8*πμa*^3^). As in the case of the solid substrate, the resistive torque is smaller for the case of larger slip for any *h*/*a*. Interestingly, for rotation on the surface of a lamella, the resistive torque coefficient decreases for small *h*/*a* to a very low value for the colloids with a relatively large slip (*λ*/*a* = 0.05).

The normalized cross coefficients - the torque due to translation and the drag due to rotation - are plotted in Fig. 6b as function of *h*/*a* for *λ* = 0.01 and *θ* = *π*/2 and it is evident that because the opposite interface is stress free and the hydrodynamic interaction is reduced, these coefficients (for a given *h*/*a*) are smaller than those for colloids on the interface of a semi-infinite liquid. The figure also shows that the two numerically computed coefficients are approximately equal; their equality is required by the reciprocity theorem.

### Hydrodynamics of a Sphere On the Surface of a Semi-infinite Liquid Layer

**Figure 7:**
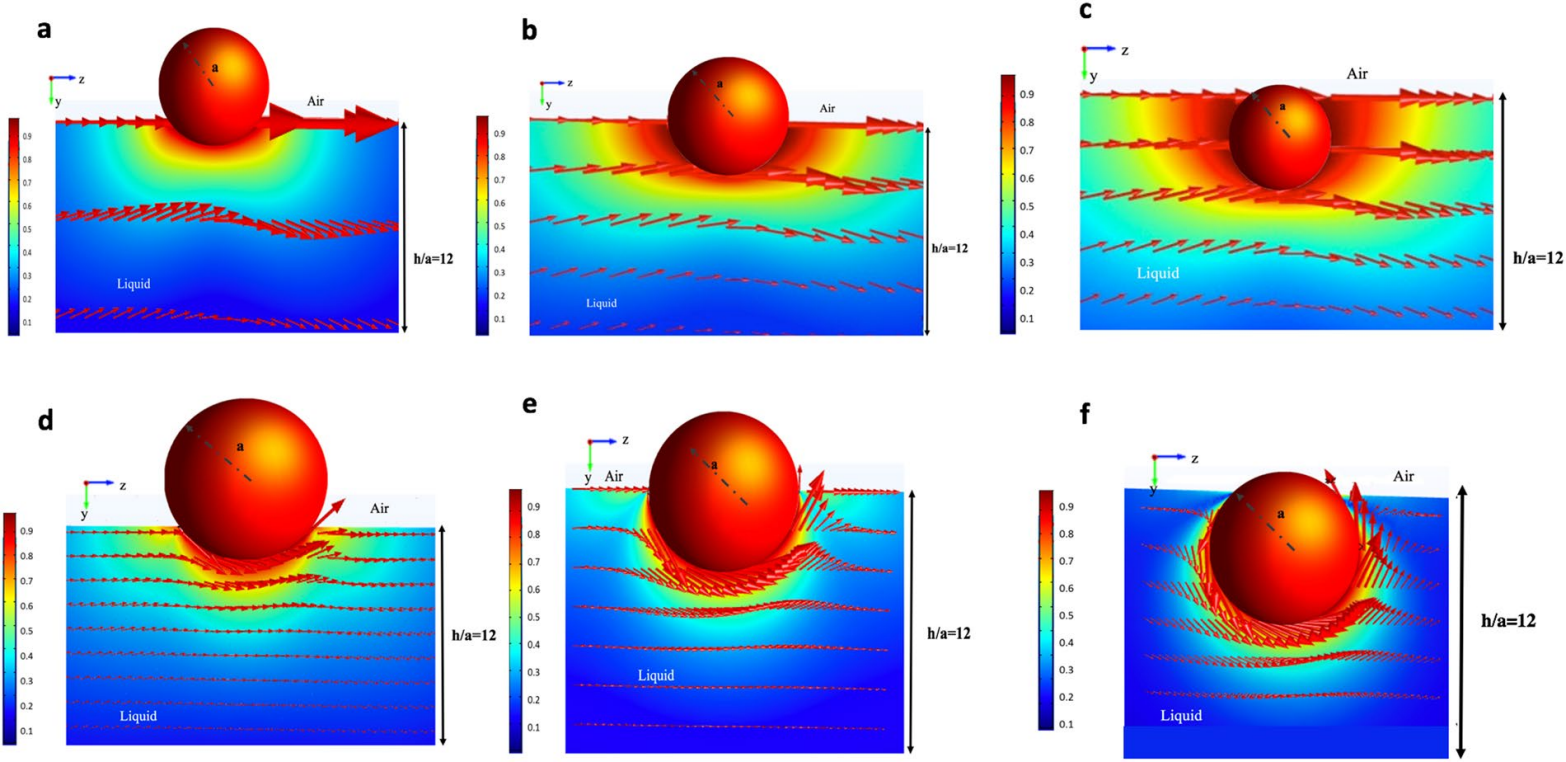
(**a**) Streamlines and velocity maps for the motion in the *x* = 0 symmetry plane for a colloid translating in the positive *z* direction with velocity *U* on the surface of a semi-infinite liquid in the laboratory frame for *λ*/*a* = 0.01 and (**a**) a hydrophobic (*θ* = 7*π*/8, *d*/*a* = 0.2), (**b**) a neutral (*θ* = *π*/2, *d*/*a* = 1) and (**c**) a hydrophilic *θ* = *π*/8, *d*/*a* = 1.8) colloid assuming the liquid to be water. Streamlines and velocity maps for the motion of the fluid underneath a colloid rotating counterclockwise around the *x* axis with velocity *a*Ω on the surface of a semi-infinite liquid in the laboratory frame for *λ*/*a* = 0.01 and (**d**) a hydrophobic rotating colloid (*θ* = 7*π*/8), (**e**) a neutrally wetting (*θ* = *π*/2 colloid and (**f**) a hydrophilic (*θ* = *π*/8) colloid.

In this section. the hydrodynamic drag and torque exerted on a translating and rotating spherical colloid straddling an air/liquid interface above a semi-infinite liquid have been calculated, and the effect of the immersion depth (contact angle) on the hydrodynamics has been studied which in the calculations in the prior sections was fixed at *θ* = *π*/2 or *d*/*a* = 1. From the asymptotic behavior of the drag or torque coefficients as a function of *h*/*a* (e.g. Figs 4 or 6) it is clear that the semi-infinite drag and torque coefficients are achieved for *h*/*a* approximately equal to 10 or larger, and here the film thickness has been fixed to 35 for semi-infinite conditions. Assuming that the liquid underlying the interface is an aqueous phase, Fig. 7a–c demonstrate the flow field around a hydrophobic (*θ* = 7*π*/8, *d*/*a* = 0.2), neutral (*θ* = *π*/2, *d*/*a* = 1) and hydrophilic *θ* = *π*/8, *d*/*a* = 1.8 colloid, respectively, translating along the interface with velocity *U* in the positive *z* direction in the laboratory frame with slip coefficient *λ*/*a* = 0.01. As would be expected, the more hydrophilic the colloid is, the greater its penetration in the liquid phase and larger is the hydrodynamic disturbance and the net viscous stresses exerted on the colloid.

**Figure 8:**
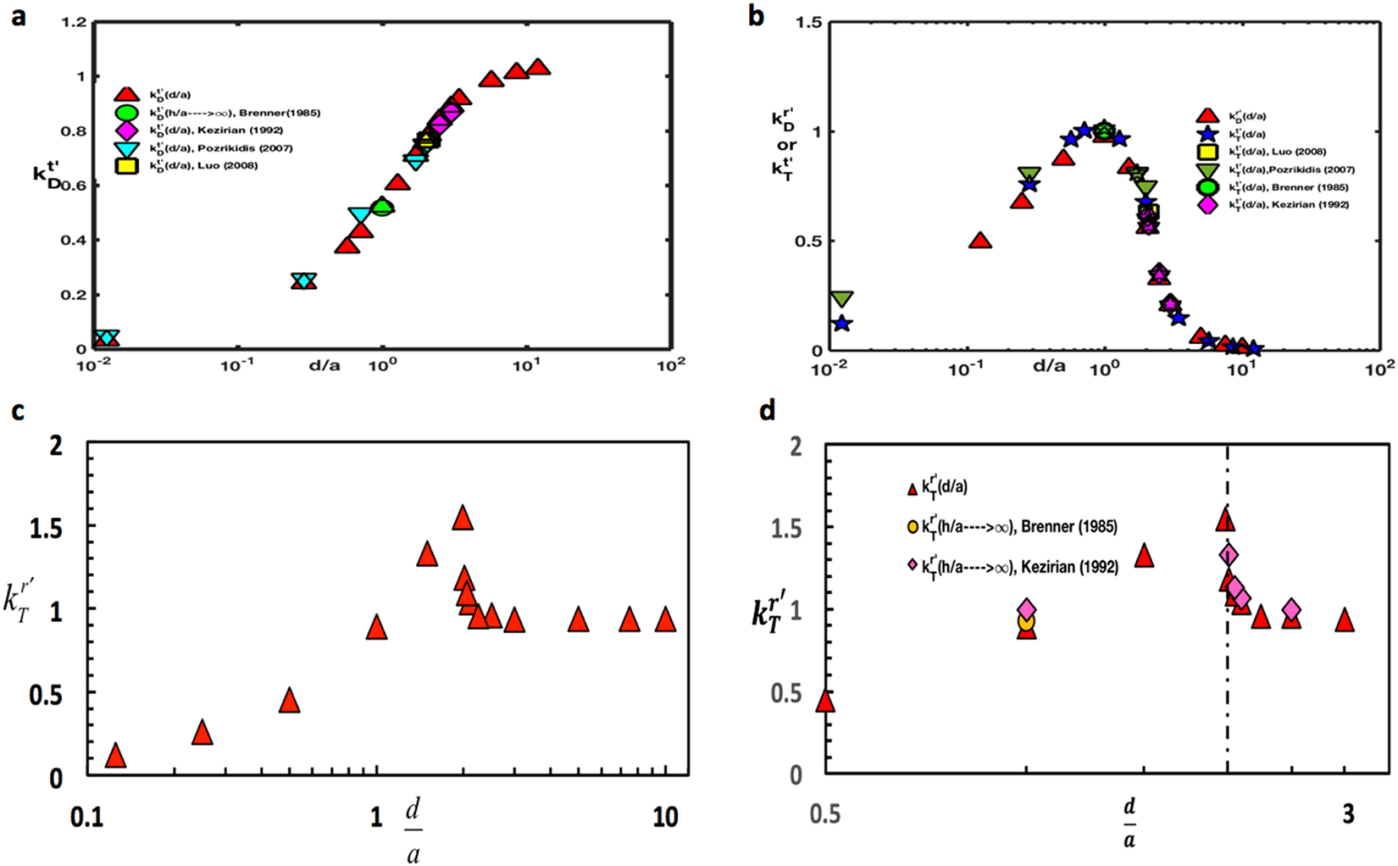
(**a**) Normalized drag coefficient (${k}_{D}^{t\text{'}}$) as function of immersion depth *d*/*a* for a translating sphere atop a semi-infinite domain, (**b**) Normalized cross coefficients as function of immersion depth for a sphere either translating or rotating atop a semi-infinite domain (**c**) normalized resistive torque coefficient (${k}_{T}^{r\text{'}}$) as function of immersion depth for a rotating sphere above a semi-infinite domain (**d**) normalized resistive torque coefficient as a function of the immersion depth in the vicinity of the interface. In a-d, the slip coefficient *λ*/*a* = 0.01 and a comparison with literature values is shown.

In Fig. 8a, the normalized drag coefficient ${k}_{D}^{t\text{'}}$ has been plotted as a function of the immersion depth of the colloid into the liquid, *d*/*a*. For immersion into a semi-infinite liquid, the drag coefficient is normalized by the Stokes drag on a colloid which is completely immersed (i.e. $6\pi \mu a\tfrac{1+2\lambda /a}{1+3\lambda /a}$). ${k}_{D}^{t\text{'}}$ is found to increase monotonically with an increase in the immersion depth (i.e. decrease in three-phase contact angle) until the colloid becomes fully immersed, and reaches the asymptotic value of Stokes drag when *d*/*a* is of the order of 10. This semi-infinite calculation has also been undertaken in several studies in the literature, e.g. for straddling spheres (0 < *d*/*a* < 2) by Brenner *et al*.^21^ and Pozrikidis^16^ and for a sphere fully immersed in the liquid (*d*/*a* > 2)^22,30^. As evident in the figure, their results are in agreement with the prior calculations over the complete range of *d*/*a*. (Note that the literature results are for a sphere with no-slip while the present calculations are for *λ*/*a* = 0.01; the presence of a small slip does not effect the drag coefficients (and also the cross coefficients, see below). However, as a result of the stress singularity at the vicinity of contact lines which recede or advance over a solid surface under no slip-conditions, a small slip on the solid (sphere) surface has a strong effect on the resistive torques due to rotation.

The cross coefficient for the torque exerted on the colloid due to the translation (${k}_{T}^{t\text{'}}$) as a function of *d*/*a* is plotted in Fig. 8b, normalized by the maximum torque coefficient for a half-immersed (*d*/*a* = 1) colloid translating in the liquid phase. In this case, when the colloid is just immersed in the liquid, the torque exerted by the translation is very small since the wetted contact area is close to vanishing. The coefficient then increases rapidly with immersion depth until the colloid is half immersed (*d*/*a* = 1) and then starts decreasing as the colloid becomes completely immersed (1<  *d*/*a<* 2). As the torque is driven by the asymmetry in the shear stress (in the *y* direction) exerted by the liquid on the colloid due to the translation (see Fig. 1), as the immersion becomes larger than *d*/*a* = 1, the shear stress at the top and bottom of the colloid become balanced and the torque tends to zero. A zero torque coefficient, which is characteristic of the torque exerted on a translating colloid which is completely immersed is achieved for *d*/*a* ≈ 10. The other cross coefficient, i.e. the normalized drag due to rotation (${k}_{D}^{r\text{'}}$, normalized with corresponding maximum) is also plotted in Fig. 8b, and is equal to ${k}_{T}^{t\text{'}}$ as required by the reciprocity condition.

The pure rotation of the colloid as a function of the immersion depth presents some interesting results concerning the effect of changing the contact angle (immersion depth) on the resistive torque exerted by the liquid on the colloid. Figure 7d–f show the streamlines for the hydrophobic, neutrally wetting and hydrophilic colloids, respectively. It is clear that for the large contact angle of the hydrophobic colloid, *θ* = 7*π*/8, the streamlines are less curved in the immediate vicinity of the contact line and the velocity gradients are therefore much smaller than the prior cases of neutrally wetting colloid rotating on the surface of thin films atop solid substrates or free lamellae (see Figs 2c and 5d, respectively), and the cases of neutrally wetting and hydrophilic colloids rotating on the surfaces above a semi-infinite liquid (Fig. 7e,f).

The normalized resistive torque due to pure rotation of the colloid, ${k}_{T}^{r\text{'}}$ nondimensionalized by the resistive torque when the colloid is completely immersed in the liquid, $\tfrac{8\pi \mu {a}^{2}}{1+3\lambda /a}$, is plotted in Fig. 8c. The resistive torque increases as the immersion depth increases (contact angle decreases) due to the larger velocity gradients which develop as the cusp angle in the contact line region becomes smaller. This increase in the local velocity gradients in the contact line region is also clear in the streamline figures (Fig. 7d–f), and the elevation in the gradients raises the viscous dissipation accounting for the increased resistive torque. The resistive torque reaches a maximum when the immersion depth approaches 2 and the contact angle approaches zero, i.e. when the colloid is highly hydrophilic. For this case, the colloid is (from below) just touching the interface. The resistive torque is finite because the surface of the colloid has finite slip (*λ*/*a* = 0.01). As the immersion depth increases beyond 2, the contact line disappears and its contribution to the resistive torque vanishes as well. Because of this, the resultant resistive torque for *d*/*a* > 2 is due only to the viscous drag around the rotating colloid. As the now completely immersed colloid descends further into the liquid phase ($d/a\geqslant 2$) the resistive torque decreases until the Stokes value for rotation in an infinite liquid is reached. Because of the assumed slip on the colloid surface, the resistive torque is continuous through the point at which the colloid breaches through the interface (i.e. *d*/*a* = 2). Plotted in Fig. 8d is the resistive torque calculated by Davis *et al*.^22^, who obtained solutions for $d/a\geqslant 2$ using eigenfunction expansions in bispherical and tangent sphere coordinates for the case in which the colloid interface has *zero* slip. For this case, the resistive torque becomes infinite as the colloid just touches the interface from the completely immersed side due to the stress singularity at the contact line as liquid advances on one side and recedes on the opposite side of the rotating sphere. The resistive torque remains infinite as the colloid straddles the interface (0 < *d*/*a* < 2). Figure 8d makes clear that as *d*/*a* tends to 2 from the liquid side, the computed coefficients are smaller than those of Davis *et al*.^22^ because of the assumption of a small but finite slip (*λ*/*a* = 0.01). Far enough from the interface in the liquid (*d*/*a* = 2.1) or a gap separation distance of one-tenth of a radius, the effect of the finite and small slip is not important and the calculations agree with the no-slip calculations of Davis *et al*.^22^.

## Discussion and Conclusions

In this study, a finite element numerical scheme (implemented by COMSOL) is being used to obtain solutions for the flow field for a spherical colloid (radius *a*) translating and rotating along a planar interface atop either (i) a thin liquid film on a solid support, (ii) a thin free liquid lamellae or (iii) a semi-infinite liquid phase (see Fig. 1a). Inertial effects were neglected so that the flow in the liquid was a Stokes flow. The air/liquid interface atop the thin liquid layers, or the semi-infinite liquid phase, was assumed to be flat up to the contact line of the colloid straddling the interface, as capillary forces were assumed larger than viscous forces so that the interface remains undeformed. With these assumptions, the flow was decomposed into realizations in which the colloid either only translates or only rotates on the surface (Fig. 1b), and the drag and torque coefficients due to the motion for each of these cases were obtained from the hydrodynamic solutions. For colloids straddling the interface of thin liquid films, the case of neutral wetting with the colloid half immersed in the liquid (i.e. the immersion depth *d* is equal to *a* or the contact angle is *π*/2) is of primary interest. The drag exerted on a translating colloid was found to be a strong function of the thickness of the film relative to the colloid radius (*h*/*a*), and whether the opposite face of the film was solid boundary or an air/liquid interface. For a colloid translating on an interface of a liquid film above a solid substrate, the drag coefficient (${k}_{D}^{t}$) *increases* significantly as the film thickness decreases to the order of the colloid radius (*h*/*a* → 1) due to the large velocity gradients (and hence large viscous lubrication forces) as a result of the strong hydrodynamic interaction of the moving colloid with the stationary bottom wall (Fig. 4a). For a colloid translating on an interface of a free lamella, the drag coefficient *decreases* as (*h*/*a* → 1) because the mobility of the bottom free interface does not allow strong velocity gradients to develop (Fig. 6a). To study the hydrodynamics of the rotation, the colloid surface was assumed to have finite but small slip (characterized by the Navier slip coefficient *λ* which was set to 0.01a). For a colloid with a no-slip surface which straddles the surface and rotates, viscous stresses become infinite along the contact line since this boundary moves relative to the surface. Hence the net resistive torque exerted on the colloid by the fluid becomes infinite and the colloid with a no-slip surface cannot rotate. Even for this small slip coefficient, the resistive torque exerted by the liquid on the rotating colloid was dominated by the stresses developed at the contact line as the coefficient for the resistive torque (${k}_{T}^{r}$) was not a strong function of *h*/*a* (Figs 4c and 6c). In the case of a colloid on the interface above a semi-infinite liquid layer, the influence of the immersion depth was studied. For translation the drag coefficient increased monotonically and continuously as the colloid becomes more immersed in the liquid (0 < *d*/*a* < 2) gradually experiencing the Stokes drag for a colloid in an infinite medium for *d*/*a* of order 10 (Fig. 8a). The increase in resistance results from the greater immersion into the liquid, and the resultant increase in the viscous traction. For rotation the resistive torque increases with immersion up to the point at which it becomes just completely immersed (*d*/*a* = 2). This increase results from the development of the large viscous stress at the contact line as the contact angle decreases and the increasingly smaller wedged shape region creates increasingly large velocity gradients (Figs 7d–f and 8c). As the colloid becomes completely immersed in the liquid, the resistive torque coefficient begins to decrease since the contact line contribution is removed and the torque coefficient decreases to the value in an infinite medium.

**Figure 9:**
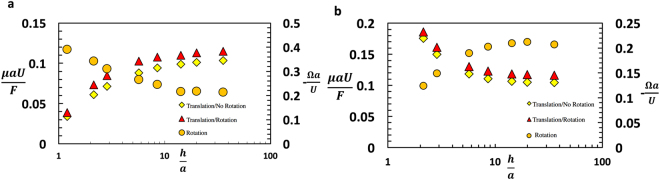
Velocity *U* and rotation Ω for a colloid attached to an air/liquid interface and subject to an external force F along the surface as in Fig. 1a for (**a**) a film on a solid support and (**b**) a free liquid film. The colloid is assume to be half-immersed at the interface (*d*/*a* = 1) and the slip coefficient is *λ*/*a* = 0.01.

The differences in the behavior of the drag and torque coefficients for colloids on the surfaces of solid-supported liquid films as opposed to free lamellae as the film thickness decreases leads to a fundamental difference in the dependence of the colloid translational velocity *U* and rotational velocity Ω when subject to an external force *F* (Fig. 1a). The force balance along the surface equates the external force to the drag due to the translation and rotation of the colloid ($F-\mu a{k}_{D}^{t}U-\mu {a}^{2}{k}_{D}^{r}{\rm{\Omega }}=0$), and the torque balance around the axis parallel to the interface and perpendicular to the motion equates the torque generated by the translation which causes the colloid to rotate with the viscous resistive torque due to rotation of the colloid (−$\mu {a}^{2}{k}_{T}^{r}{\rm{\Omega }}-\mu a{k}_{T}^{t}U=0$). Thus $\frac{a{\rm{\Omega }}}{U}=-\,\frac{{k}_{T}^{t}}{{k}_{T}^{r}}$ and $U=\frac{F}{\mu a{k}_{D}^{t}}{(1-\frac{{({k}_{T}^{r})}^{2}}{{k}_{T}^{r}{k}_{D}^{t}})}^{-1}$. As clear in Fig. 9a, velocity of the sphere decreases with a decrease in the film thickness of a solid supported film due to increased hydrodynamic interaction and increased drag. The sliding coefficient $\frac{{\rm{\Omega }}a}{U}$ increases with a decrease in film thickness as the torque due to translation increases and the colloid tends to roll more than slide. This behavior has applications in the use of colloids adsorbed to the interfaces of thin liquid layers on solid substrates to form colloid surface coatings by evaporative assembly driven by capillary attraction forces. As the film thickness decreases due to evaporation, as shown in 9a for a constant force the approach velocities of the colloids due to the hydrodynamic interaction with the wall would decrease, so the assembly process slows down. However, as 9a indicates, the motion at the later stages of assembly would be more rolling then sliding (Ωa/U increases) and this can be important if the orientations of the assembling colloids is required to be preserved.

Opposite effects are observed (Fig. 9b) when the opposite interface is a free liquid interface as the drag due to liquid layer increases with increase in film thickness and colloid velocity is more as the film thins out. The colloid tends to roll more than slide as the film becomes thicker. For both cases, the regime in which the colloid is not allowed to rotate (i.e. the contact angle singularity arrests the motion, $\lambda \to 0,\,{k}_{T}^{r}\to {\rm{\infty }}$ have also been considered. For this no slip case, the translational velocities of the colloid on the surface of a film atop a solid or a free film become reduced relative to the case where it is permitted to rotate.

## Methods

As shown in Fig. 1, because the motion is assumed to be at low Reynolds number, the flow is governed by the Stokes equations of motion which are linear in the fluid velocity. The boundary conditions on the velocity are also linear, and the free interface positions are planar and fixed. Therefore, the translational and rotational motion of the colloid can be expressed as the sum of a purely translational motion and a purely rotational one. The equations (and boundary conditions) for each of these two motions are formulated and solved separately, and the drag and torque coefficients are obtained from these solutions. When an applied lateral force acts on the colloid to drive the translation and rotation, the translational and angular velocities are obtained by computing the net hydrodynamic drag and torque on the colloid using the coefficients obtained from the two flow realizations. (see the Discussion and Conclusion section of the study). Here we briefly outline the governing equations and boundary conditions for each of the two problems. The mass conservation (incompressibility constraint) and the Stokes equations take the form:

1 $$-\nabla p+{\nabla }^{2}{\bf{v}}=0,$$

2 $$\nabla \cdot {\bf{v}}=0,$$

where ${\nabla }^{2}$ is the Laplacian, **v** is the local fluid velocity field non-dimensionalized by *U* the velocity of the colloid (or Ωa), *p* is the pressure normalized by $\frac{\mu U}{a}$ (or *μ*Ω), where *μ* is the viscosity of the liquid.

For both problems, at the planar top free interface (i.e. the air/liquid surface), the velocity normal to the surface (*y* direction) is zero and the tangential shear stress (*x* and *z* directions) is zero, $({\bf{I}}-{{\bf{n}}}_{f}{{\bf{n}}}_{f})\cdot \{\nabla {\bf{v}}+{(\nabla {\bf{v}})}^{\dagger }\}\cdot {{\bf{n}}}_{f}={\bf{0}}$ where **n**_*f*_ is the normal vector to the upper air/liquid interface, ${{\bf{n}}}_{f}={\hat{{\bf{e}}}}_{y}$ and the gradients in fluid velocity are evaluated at the fluid interface. Similarly for the bottom surface, if the surface is a solid the velocity is required to be zero. If the bottom surface is a free interface, the normal velocity (*y* direction) and the tangential shear stresses are zero. The bounded computational domain is detailed in the Supplementary information (see Fig. A1a), and the domain is taken large enough in the *x* and *z* directions so that the results are independent of these bounds. At the bounding sides of the computational domain which are parallel to the *x* axis, the velocity is required to be zero. For the bounding surfaces in the *z* direction, which represent the flow direction for both motions, the upstream velocity is set equal to zero and the downstream pressure is set equal to zero.

At the surface of the solid colloid, the kinematic boundary conditions are formulated to include slip at the interface, as is required to allow the colloid to rotate. In the case of translation, the *colloid* velocity (nondimensionally) is equal to ${\hat{{\bf{e}}}}_{z}$, where ${\hat{{\bf{e}}}}_{z}$ is the unit vector in the *z* direction (see Fig. 1). The normal component of this velocity is equal to the fluid velocity at the surface (${{\bf{v}}}_{s}$), i.e. ${\bf{n}}\cdot ({\hat{{\bf{e}}}}_{z}-{{\bf{v}}}_{s})=0$, where **n** is the local (outward) normal to the colloid. Denoting by **t** a unit tangent vector to the colloid surface and ${{\boldsymbol{\tau }}}_{s}={\rm{\nabla }}{\bf{v}}+{\rm{\nabla }}{{\bf{v}}}^{\dagger }$ the (nondimensional) viscous stress tensor evaluated at the surface, the slip condition becomes:

3 $${\boldsymbol{t}}\cdot ({\hat{{\bf{e}}}}_{z}-{{\bf{v}}}_{s})=\frac{\lambda }{a}({\bf{n}}\cdot {{\boldsymbol{\tau }}}_{s}\cdot {\bf{t}})$$

Note that this tangential slip condition is formulated for two orthogonal tangential directions on the surface.

For the purely rotational case, the (dimensionless) rotational velocity vector of the surface is ${\hat{{\bf{e}}}}_{x}$ where ${\hat{{\bf{e}}}}_{x}$ is the unit vector in the *x* direction (see Fig. 1) and the tangential velocity is given by ${\hat{{\bf{e}}}}_{x}\times ({{\bf{x}}}_{p}-{{\bf{x}}}_{o})$ where ${{\bf{x}}}_{p}$ and ${{\bf{x}}}_{o}$ are position vectors on the colloid surface and center, respectively (scaled with *a*). The normal component of the fluid velocity at the surface is zero (**n** · **v**_*s*_ = 0), and the tangential slip condition is given by

4 $${\boldsymbol{t}}\cdot ({\hat{{\bf{e}}}}_{x}\times ({{\bf{x}}}_{p}-{{\bf{x}}}_{o})-{{\bf{v}}}_{s})=\frac{\lambda }{a}({\bf{n}}\cdot {{\boldsymbol{\tau }}}_{s}\cdot {\bf{t}})$$

The hydrodynamic flow generates a traction and torque on the surface of the translating or rotating colloid, and for each flow realization these are given (in nondimensional form) by:

5 $${{\bf{F}}}_{P}=\mathop{\oiint }\limits_{{{\rm{\Gamma }}}_{P}}\,({\boldsymbol{\sigma }}\cdot {\bf{n}})\,ds,$$

6 $${{\bf{T}}}_{P}=\mathop{\oiint }\limits_{{{\rm{\Gamma }}}_{P}}\,({{\bf{x}}}_{p}-{{\bf{x}}}_{o})\times ({\boldsymbol{\sigma }}\cdot {\bf{n}})\,ds.$$

where Γ_*P*_ denotes the portion of the colloid surface in contact with the liquid, and ${\boldsymbol{\sigma }}=-p{\bf{I}}+{\rm{\nabla }}{\bf{v}}+{\rm{\nabla }}{{\bf{v}}}^{\dagger }$ is the total stress tensor. From the computed traction and torque, the drag and torque coefficients are computed.

The hydrodynamic equations and boundary conditions for each of the flow realizations were solved using the COMSOL Multiphysics numerical software which uses the finite element method to solve the field equations and boundary conditions. Briefly, in this technique, the variables are discretized as sums over a set of basis (approximating) functions defined over local spatial domains (three dimensional elements) for which the computational domain is partitioned, and the tessellation of the domain is designed to merge smoothly with the boundaries of the domain.

## Electronic supplementary material

Supplementary Information

## References

[1] Abbott, E. A. *Flatland: A romance of many dimensions* (OUP Oxford, 2006).

[2] Wu, J. & Ma, G.-H. Recent studies of pickering emulsions: Particles make the difference. *Material Reviews* (2016). 10.1002/smll.201600877 27337222

[3] Bournival G, Ata S, Wanless EJ (2015). The roles of particles in multiphase processes: Particles on bubble surfaces. Advances in Colloid and Interface Science.

[4] Prevo BG, Kuncicky DM, Velev OD (2007). Engineered deposition of coatings from nano- and micro-particles: A brief review of convective assembly at high volume fraction. Colloids and Surfaces A: Physicochemical and Engineering Aspects.

[5] Lotiti V, Zambelli T (2017). Approaches to self-assembly of colloidal monolayers: A guide for nanotechnologists. Advances in Colloid and Interface Science.

[6] Fei W, Gu Y, Bishop K (2017). Active colloidal particles at fluid-fluid interfaces. Current Opinion in Colloid and Interface Science.

[7] Dorr A, Hardt S (2015). Driven particles at fluid interfaces acting as capillary dipoles. Journal of Fluid Mechanics.

[8] Vassileva ND, van den Ende D, Mugele F, Mellema J (2005). Capillary forces between spherical particles floating at a liquid-liquid interface. Langmuir.

[9] Dalbe M-J, Cosic D, Berhanu M, Kudrolli A (2011). Aggregation of frictional particles due to capillary attraction. Physical Review E.

[10] Boneva MP, Christov NC, Danov KD, Kralchevsky PA (2007). Effect of electric-field-induced capillary attraction on the motion of particles at an oil–water interface. Physical Chemistry Chemical Physics.

[11] Boneva MP, Danov KD, Christov NC, Kralchevsky PA (2009). Attraction between particles at a liquid interface due to the interplay of gravity-and electric-field-induced interfacial deformations. Langmuir.

[12] Dani A, Keiser G, Yeganeh M, Maldarelli C (2015). Hydrodynamics of particles at an oil-water interface. Langmuir.

[13] Cheung D (2010). Molecular simulation of nanoparticle diffusion at fluid interfaces. Chemical Physics Letters.

[14] Koplik J, Maldarelli C (2017). Diffusivity and hydrodynamic drag of nanoparticles at a vapor-liquid interface. Phys. Rev. Fluids.

[15] Fischer TM, Dhar P, Heinig P (2006). The viscous drag of spheres and filaments moving in membranes or monolayers. Journal of Fluid Mechanics.

[16] Pozrikidis C (2007). Particle motion near and inside an interface. Journal of Fluid Mechanics.

[17] Danov K, Aust R, Durst F, Lange U (1995). Influence of the surface viscosity on the drag and torque coefficients of a solid particle in a thin liquid layer. Chemical Engineering Science.

[18] Danov KD, P. B, Dimova R (2000). Viscous drag of a solid sphere straddling a spherical or flat surface. Physics of Fluids.

[19] Dörr A, Hardt S, Masoud H, Stone HA (2016). Drag and diffusion coefficients of a spherical particle attached to a fluid–fluid interface. Journal of Fluid Mechanics.

[20] Vidal A, B. L (2017). Slip flow past a gas–liquid interface with embedded solid particles. Journal of fluid mechanics.

[21] O’Neill ME, B. H, Ranger KB (1985). Slip at the surface of a translating-rotating sphere bisected by a free surface bounding a semi infinite viscous fluid: Removal of the contact line singularity. Physics of Fluids.

[22] Anthony MJ, Davis HB, Kezirian MichaelT (1994). On the stokes-einstein model of surface diffusion along solid surfaces: Slip boundary conditions. Journal of Colloid and Interface Science.

[23] Snoeijer JH, Andreotti B (2013). Moving contact lines: scales, regimes, and dynamical transitions. Annual review of fluid mechanics.

[24] Neto C, Evans DR, Bonaccurso E, Butt H-J, Craig VSJ (2005). Boundary slip in newtonian liquids: a review of experimental studies. Reports on Progress in Physics.

[25] Lauga, E., Brenner, M. & Stone, H. *Microfluidics: The No-slip Boundary Condition*, chap. 19, 1219–1240 (Springer, 2007).

[26] Bocquet L, Charlaix E (2010). Nanofluidics, from bulk to interfaces. Chem. Soc. Rev..

[27] Lee T, Charrault E, Neto C (2014). Interfacial slip on rough, patterned and soft surfaces: A review of experiments and simulations. Advances in Colloid and Interface Science.

[28] Hood K, Lee S, Roper M (2015). Inertial migration of a rigid sphere in three-dimensional poiseuille flow. Journal of Fluid Mechanics.

[29] Chan PC-H, L. GL (1977). A note on the motion of a spherical particle in a general quadratic flow of a second-order fluid. Journal of Fluid Mechanics.

[30] Luo H, Pozrikidis C (2008). Effect of surface slip on stokes flow past a spherical particle in infinite fluid and near a plane wall. Journal of Engineering Mathematics.

