# Supplementary material for: The Translational and Rotational Dynamics of a Colloid Moving Along the Air-Liquid Interface of a Thin Film
Source: Sci Rep. 2018 Jun 11;8:8910. doi: 10.1038/s41598-018-26121-0 (PMC5995853; doi:10.1038/s41598-018-26121-0)
Supplement: Supplementary file 1 — Supplementary Information [file 41598_2018_26121_MOESM1_ESM.pdf]

Supplementary Material for "The Translational and Rotational Dynamics of a Colloid Moving Along the Air-Liquid Interface of a Thin Film"

Subhabrata Das[1], Joel Koplik[2], Raymond Farinato[3], D.R. Nagaraj[3], Charles Maldarelli[4],and Ponisseril Somasundaran[1]
[1] *Columbia University, Langmuir Center of Colloids and Interfaces at New York, 10025, USA*
[2] *City College of The City University of New York, Levich Institute and Department of Physics, New York, 10027, USA*
[3] *Solvay Technology Solutions, Stamford, USA*
[4] *City College of The City University of New York, Levich Institute and Department of Chemical Engineering, New York, 10027, USA*
(Dated: May 15, 2018)

MESH CONVERGENCE DETAILS

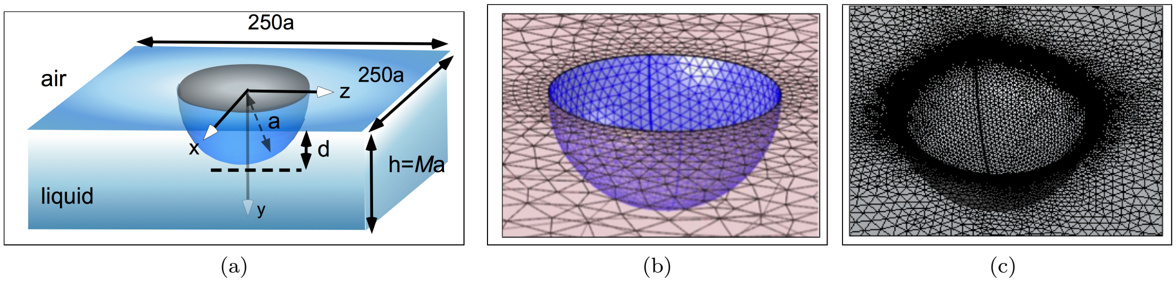

FIG. 1: (a) Schematic representation of the computational domain, b) fully resolved mesh for a translating sphere and (c) rotating sphere in the vicinity of the contact line for converged solutions for h/a=35 (semi-infinite case), $\theta = \pi/2$ and $\lambda/a = .01$

A solid spherical colloid with radius a, translating along the z axis or rotating around the x axis at the origin of Cartesian coordinate system is located in the computational volume of a rectangular box of dimensions $250a(in\ \mathrm{z}) \times Ma(in\ \mathrm{y}) \times 250a(in\ \mathrm{x})$ where M varies from $1.2-35$ to account for different film thicknesses and to achieve semi-infinite conditions [see Fig. 1a]. The values used for the z and the x dimensions insured that the results did not depend on the lateral dimensions of the box (i.e. the interface was infinite in extent). Reduction of the box size to $200a \times 200a$ resulted in values of the drag and torque coefficients that changed from the $250a \times 250a$ results by less than a few percent.

Numerical solutions for the fluid flow in the computational domain are obtained by using the COMSOL Multiphysics program, which solves the mass conservation and hydrodynamic equations and associated boundary conditions (see Methods) using a finite element method with a mesh discretization of the computational domain that is built using a custom construction feature of the software. The computational domain is discretized into tetrahedral elements by first applying triangular meshes to the surfaces of the computational domain and then subdividing the volume into tetrehedra that connect to the triangular surface elements. The lengths of the sides of the triangular elements on the section of the surface of the sphere within the computational domain and the lengths of these surface elements which intersect the contact line edge are the key parameters for assigning specific numerical values for the lengths of the elements of the mesh construction, and for the refinement of the mesh to achieve converged solutions. In general, the range (maximum and minimum sizes) for the lengths of the surface (η_{max}/a and η_{min}/a, respectively) and contact line edge elements (ξ_{max}/a and ξ_{min}/a) are specified, and the remainder of the surface element lengths and the edge lengths are assigned by the program using their default normal construction. After a solution is obtained, drag and torque coefficients are computed by integration. The convergence criterion is set to 0.0001% relative error /tolerance) and it took 16 iterations to reach convergence. An iteration scheme is set-up in which the size parameters for the surface and edge elements are reduced and the coefficients recomputed until the recomputed values differ by less than two percent from the previous iteration.

For converged solutions, the mesh refinement needed in the case of rotation was much finer than for translation. In Table I are given, for a translating sphere, the parameters for the last two iterations in the refinement that resulted in convergence. These parameters correspond to the case in which the contact angle is $\pi/2$, the film thickness (h) divided

by the radius (a) is equal to 35, which is large enough to obtain the results for translation above a semi-infinite liquid. This particular calculation can be checked against the Brenner et al[1] analytical solution which allows verification of the numerical solution. Table I shows that the error relative to the analytical solution is less than approximately five percent, and in Fig. 1b the triangular surface meshes at convergence are shown.

λ /a	η_{max}/a	η_{min}/a	ξ_{max}/a	ξ_{min}/a	k_D^t	k_T^t	% Error in k_D^r	% Error in k_T^r
0.01	0.1	0.01	0.05	0.005	9.673	4.7121	2.68	2.99
0.01	0.09	0.009	0.05	0.005	9.563	4.6582	1.518	1.82

TABLE I: Convergence of drag and torque coefficients for translating sphere with mesh refinement for h/a=35, $\theta = \pi/2$ and $\lambda/a = .01$.

For rotational motion, a much finer mesh is required on the surface of the sphere at the contact line due to the large velocity gradients at the line, and η_{max}/a, η_{min}/a, ξ_{max}/a and ξ_{min}/a are reduced considerably to achieve convergence and agreement with the analytical calculation (Table II and Fig. 1c). We found, as noted by Yulil et al [2], that fine mesh parameterization also removed spurious vortices near the corners of the rotating sphere in order to obtain accurate values in particular for the torque coefficient due to rotation. A comparison of the number of mesh

λ/a	η_{max}/a	η_{min}/a	ξ_{max}/a	ξ_{min}/a	k_D^r	k_T^r	% Error in k_D^r	% Error in k_T^r
0.01	0.05	0.0005	0.02	0.0002	4.9989	20.459	11.92	9.265
0.01	0.035	0.00035	0.02	0.0002	4.3797	24.059	3.57	4.269
0.01	0.02	0.0002	0.02	0.0002	4.3867	24.021	4.116	3.41
0.05	0.05	0.0005	0.02	0.0002	4.3381	15.438	5.85	4.64
0.05	0.04	0.0004	0.03	0.0003	4.2429	15.163	3.535	6.343

TABLE II: Convergence of drag and torque coefficients for rotating sphere with mesh refinement for h/a=35, $\theta = \pi/2$ and λ/a = .01 and .05.

vertices, the number of tetrahedral volume elements, surface triangular and edge elements are given in Table III .

Mesh Parameter	Translating Sphere	Rotating Sphere
Number of Mesh Vertices	7537	51772
Number of Tetrahedral Elements	36990	241295
Number of Triangular Elements	5154	48196
Number of Edge Elements	272	1179

TABLE III: Mesh Statistics for a translating and rotating sphere at interface of thin film on a solid substrate for h/a=35, $\theta = \pi/2$ and $\lambda/a = .01$

Finally, in the cases where the distance between the bottom interface and the bottom of the sphere surface or (for complete immersion) the top free surface and the top of the sphere become of the order of the radius of the sphere finer meshes were constructed in this gap by decreasing the lengths of the edge elements for the tetrehedra and gas/liquid and liquid/solid surface triangular elements. We note that as the mesh around the sphere becomes finer, inversion of the finite element matrix becomes more difficult and leads to longer computational times.

[1] H. B. M. E. O'Neill, K. B. Ranger, Physics of Fluids **29**, 913 (1985).
[2] Y. D. Shikhmurzaev, Physica D: Nonlinear Phenomena **217**, 121 (2006).
